# SDS-PAGE Protein and HPTLC Polyphenols Profiling as a Promising Tool for Authentication of Goldenrod Honey

**DOI:** 10.3390/foods11162390

**Published:** 2022-08-09

**Authors:** Małgorzata Dżugan, Michał Miłek, Patrycja Kielar, Karolina Stępień, Ewelina Sidor, Aleksandra Bocian

**Affiliations:** 1 Department of Chemistry and Food Toxicology, Institute of Food Technology and Nutrition, University of Rzeszow, Ćwiklińskiej 1a, 35-601 Rzeszow, Poland; 2 Department of Biology, Institute of Biology and Biotechnology, University of Rzeszow, Zelwerowicza 4, 35-601 Rzeszow, Poland; 3 Doctoral School, University of Rzeszow, Rejtana 16c, 35-959 Rzeszow, Poland; 4 Department of Biotechnology and Bioinformatics, Faculty of Chemistry, Rzeszow University of Technology, Powstańców Warszawy 6, 35-959 Rzeszow, Poland

**Keywords:** antibacterial activity, antifungal activity, authentication, goldenrod, honey, protein, polyphenols

## Abstract

The aim of the study was to use protein and polyphenolic profiles as fingerprints of goldenrod honey and to apply them for verification of the labeled variety. The markers for 10 honey samples were correlated with the standard physicochemical parameters and biological activity measured in vitro as antioxidant, antifungal and antibacterial activities. Honey proteins were examined regarding soluble protein, diastase and SDS-PAGE protein profile. The polyphenolic profile was obtained with the use of the HPTLC and the antioxidant activity was detected with standard colorimetric methods. The antimicrobial effect of representative honey samples of different chemical profiles was verified against *E. coli* and budding yeast. It was found that the SDS-PAGE technique allows for creating the protein fingerprint of the goldenrod honey variety which was consistent for 70% of tested samples. At the same time, the similarity of their polyphenolic profile was observed. Moreover, specific chemical composition resulted in higher bioactivity of honey against tested bacteria and yeast. The study confirmed the usefulness of both SDS-PAGE and HPTLC techniques in honey authentication, as an initial step for selection of samples which required pollen analysis.

## 1. Introduction

Among nectar honeys, which differ in terms of properties and taste, goldenrod honey has recently become more and more popular. It is related to the growing supply of goldenrod as a honeyflow in Central and Eastern Europe, including Poland. On the other hand, goldenrod honey is still one of the less-studied varieties, and few literature data from recent years concern the chemical profiles and antioxidant properties of this honey [1,2]. Many beneficial properties of this honey are reported: antibiotic and supporting the urinary system, skin and circulatory system. However, due to the lack of confirmation of this varietal honey features in scientific literature, natural medicine attributes its healing effect to the properties of the goldenrod plant. Goldenrod (*Solidago* sp.) is a controversial plant: on the one hand, it has some phytotherapeutic effects (in diseases of the skin, respiratory system, circulation, urinary system, even in the treatment of certain cancers and depression [3,4,5], and on the other hand, popular species *S. gigantea* and *S. canadensis* are classified as invasive plants [6,7]. Only *Solidago virgaurea*, a native European species is listed in pharmacopoeias as an official herbal drug [5]. The most common species of *Solidago*, i.e., in south-eastern Poland, mainly *S. gigantea* and *S. canadensis*, are used as beeflows. Jasicka-Misiak et al. [1] refer to *S. virgaurea* as the source of nectar, but more recent research points to the remaining common species of goldenrod used by bees [2]. The availability of different species may be one of the reasons for the variability of goldenrod honeys, depending on their geographic origin.

Goldenrod honey owes its healing properties to the flowers from which it is obtained, i.e., *Solidago* spp. plants. Thanks to it, goldenrod honey contains flavonoids (including quercetin) as well as tannins and organic acids. It is rich in vitamins and bactericidal substances. It is characterized by a specific profile of volatile compounds among which germacrene D has been recognized as a marker compound of this honey variety [8]. In natural medicine, this type of honey is considered particularly valuable and recommended for its anti-inflammatory, diuretic, choleretic and diastolic effects on the genitourinary system; however, these features have been not objective of scientific research. This honey type has a characteristic, lemon-like, sour aftertaste and a pleasant aroma. It crystallizes relatively quickly, creating a creamy consistency. Goldenrod honey is available in shades of yellow to amber [2]. It becomes much brighter after crystallization. This honey variety does tend to crystallize fast in a matter of weeks [8]. Crystallization does not change the flavor or spoil the honey. Moreover, the specific feature of goldenrod honey is susceptibility to fermentation, which can occur during storage at room temperature.

Assessing the authenticity of honey is a serious problem that has gained much interest internationally because honey has frequently been subject to various fraudulent practices, including mislabeling of botanical and geographical origin and mixing with sugar syrups or honey of lower quality. Since now, melissopalynological analysis has been the only recognized analysis to confirm honey variety. However, this technique based on counting plant pollen which occurs in honey is expensive and time-consuming. To protect the health of consumers and reduce the unfair practice of honey adulteration, the different approaches used to assess the authenticity of honey, specifically by the application of advanced instrumental techniques, have been proposed. The protein and other nitrogen compounds have been rarely studied [8,9,10,11,12,13].

The aim of the study was to construct the fingerprints of goldenrod honey based on protein and polyphenolic profiles. For the first time, the SDS-PAGE protein analysis and HPTLC polyphenols profiling were used to verify the honey variety. An attempt was made to determine whether it is possible to identify the species of goldenrod that bees used for honey production.

## 2. Materials and Methods

### 2.1. Honey and Plant Material

Ten samples of honeys declared as goldenrod were purchased in the 2021 beekeeping season from apiaries located in the Podkarpackie Province (Poland).

The flowers of three species of goldenrod (*Solidago virgaurea, Solidago gigantea* and *Solidago canadensis)* were collected from natural habitats in August 2021 (50°045′ N 21°862′ E; 50°048′ N 21°869′ E; 50°039′N 21°894′ E). After botanical identification, the samples were dried in the dark, at a temperature not exceeding 40 °C. Voucher specimens were deposited in Department collection under the numbers SV/08/21, SG/08/21, and SC/08/21.

### 2.2. Extracts Preparation

Dried plant material was pulverized using pestle and mortar and weighted (5 g) into conical flasks. After flooding with 50 mL of 50% ethanol, it was extracted in an ultrasonic bath (Sonic 10, Polsonic, Warszawa, Poland) for 20 min, then filtered through a filter paper. Ethanol was evaporated using a centrifugal evaporator (VC 2–18 CDPlus, Martin Christ, Osterode am Harz, Germany) and then the aqueous residue was lyophilized using Alpha 1–2 LD plus freeze dryer (Martin Christ, Osterode am Harz, Germany).

For chromatographic analyses, honey samples were prepared using solid phase extraction (SPE). Twenty grams of each honey were dissolved in 100 mL of acidified distilled water (pH = 2) and passed through C-18 Sep Pack Cartridge (Waters Corporation, Milford, MA, USA), preconditioned with 10 mL of methanol and 10 mL of acidified (pH = 2) water. After washing the sugars with acidified water, the polyphenols were eluted with methanol (2.5 mL).

### 2.3. Water Content

The determination of water content was done by the refractometric method, using an electronic refractometer for honey HI96800 (Hanna Instruments, Woonsocket, RI, USA). The value was determined in 21 ± 2 °C. with to the second decimal place. The determinations were made in triplicates.

### 2.4. pH and Free Acidity

To determine active acidity, a pH measurement of 20% solutions of honey extract in boiled, cold distilled water was performed using a CP-401 pH meter (Elmetron, Zabrze, Poland). To determine the free acidity, 50 mL of 20% appropriate honey extracts were titrated by 0.1 M NaOH to reach a pH of 8.3 measured by pH meter. The results were expressed in mEq/100 g.

### 2.5. Conductivity

To determine the electrical conductivity, 20% solutions of honey extracts in boiled, cold distilled water were used. The conductivity of each sample was determined by immersing the electrode in the test solution. Each sample was measured three times. The conductivity of each product solution was measured using a CP-401 conductometer (Elmetron, Zabrze, Poland) and the results (in mS/cm) were presented.

### 2.6. Color Analysis

The color of the honey was analyzed using a dedicated HI 96785 colorimeter (Hanna Instruments, Woonsocket, RI, USA). The results were expressed on the Pfund scale.

### 2.7. A-Amylase Assay

α-amylase was determined by a spectrophotometric method with the Phadebas Diastase test (Magle AB, Lund, Sweden) according to the manufacturer’s instructions. The results of absorbance were measured at wavelength 620 nm against a blank (acetate buffer) using a Biomate 3 spectrophotometer (Thermo Scientific, Waltham, MA, USA). The results were calculated according to the formula attached in the manual and expressed as diastase number (DN).

### 2.8. Soluble Protein

The protein content in honey was determined using the Bradford method according to Latimer [14]. One thousand µL of Bradford reagent (G-250) was added to 20 μL of each honey sample. Samples were incubated for 5 min at room temperature and the absorbance at 595 nm was read using a Biomate 3 spectrophotometer (Thermo Scientific, Waltham, MA, USA). The results were calculated from a calibration curve 0–100 μg/sample (y = 0.0551x, r^2^ = 0. 9991). Bovine albumin has been used as the standard.

### 2.9. Total Phenolic and Flavonoids Content

Total phenolic and flavonoid content in honey and plant extracts samples were determined according to procedures described by us previously [13]. In the case of honeys, 5% solutions in distilled water were used, and in the case of plant extracts, crude extracts diluted properly were taken for analysis.

### 2.10. Antioxidant Capacity Assays

Antioxidant capacity using DPPH and FRAP methods was determined according to procedures described by us previously [13]. The CUPRAC method was applied according to Matłok et al. [15]. Briefly, 10 μL of each diluted sample was pipetted into microplate wells, then 40 µL of CuCl_2_ (10 mM), 50 µL of neocuproine (7.5 mM), and 50 µL of ammonium acetate (1 M) were added. The absorbance was measured with a microplate reader (EPOCH 2, BioTek, Winooski, VT, USA) at 450 nm after 30 min incubation in the dark against a blank. The result was expressed in Trolox equivalents (µmol TE/100 g) from the standard curve (y = 0.026x, r^2^ = 0.9973).

### 2.11. HPTLC Analyses

The analyses were performed with the use of the CAMAG (Muttenz, Switzerland) HPTLC chromatography set, consisting of a semi-automated sample applicator (Linomat 5), an automatic developing chamber (ADC 2), a derivatizer and a visualizer. The extract samples were applied on the HPTLC plate (HPTLC Silica Gel 60 F254 plates, 20 cm × 10 cm, Merck, Darmstadt, Germany) in a volume of 5 µL, as 8 mm wide bands. Two different systems for the mobile phase and derivatization reagent were used: A–mobile phase: ethyl acetate, acetic acid, formic acid, water (10:1.1:1.1:2.6) and derivatizing agent: Natural Product Reagent/Polyethylene glycol 400 (NP/PEG); B—mobile phase: chloroform, ethyl acetate, formic acid (5:4:1) and derivatizing agent: *p*-anisaldehyde reagent with heating in 110 °C (10 min). After derivatization, plates were photographed in visible light and UV (366 nm). The obtained images were analyzed using HPTLC software (Vision CATS, CAMAG, Muttenz, Switzerland).

### 2.12. Protein Profiling by SDS-PAGE

Ten samples of goldenrod honey and four reference honey samples (heather, honeydew, rape and multifloral) were prepared as in previous work with minor modifications [16]. One gram of raw honey was dissolved in 1 mL of deionized water containing 2% Nonidet P-40 substitute and 2% dithiothreitol. The samples were then mixed 2 to 1 with 4x concentrated standard Laemmli buffer and were incubated for 5 min at 95 °C. After cooling, 15 µL of the samples were applied to 15% denaturing gels (with 3% stacking gels). Electrophoresis was carried out on a Mini-Protean II apparatus (Bio-Rad Laboratories, Hercules, CA, USA) at 50 V for 15 min and then at 200 V for 1.5 h according to the standard method of Laemmli, with the BlueEasy Prestained Protein Marker (NIPPON Genetics EUROPE, Düren, Germany) as a molecular weight marker and Tris-Glycine-SDS buffer. After electrophoresis, gels were stained with Coomassie Brilliant Blue G-250. The staining was performed overnight and then gels were destained for 24 h with deionized water. Gels were scanned with Image Scanner III (GE Healthcare, Little Chalfont, UK) and processed by LabScan 6.0 (GE Healthcare, Little Chalfont, UK). Gels analysis was performed in ImageJ (1.52a) software to generate a graphical representation of each lane on the gel to assist in sample comparison. For better visualization, individual bands from the SDS-PAGE gel were contrasted with the profile obtained with the software.

### 2.13. Yeast Strain and Growth Conditions

The strain used in this study is a reference haploid wild-type yeast *Saccharomyces cerevisiae* strain BY4741 (*MATa his3Δ leu2Δ met15Δ ura3Δ*; Euroscarf, Germany). Yeast cells were grown in a standard rich liquid YPD medium (1% Difco yeast extract, 1% yeast bactopeptone, and 2% (*w/v*) glucose) on a rotary shaker at 150 rpm or on a solid YPD medium containing 2% agar. The experiments were carried out at an optimal temperature for yeast: 28 °C.

### 2.14. Kinetics of the Yeast Growth Assay

Yeast cells were grown in a liquid YPD medium (1% Difco yeast extract, 1% yeast bactopeptone, 2% (*w/v*) glucose) without (control) or with tested honeys or extracts on a rotary shaker at 150 rpm, or on a solid YPD medium containing 2% agar. The experiments were carried out at 28 °C. The growth was monitored using an Anthos 2010 type 17 550 microplate reader (Biochrom, Cambridge, UK) at 600 nm by measurements at 2 h intervals for 12 h. Each experiment was repeated at least three times.

### 2.15. Yeast Cell Viability

For determining cell death, standard staining with propidium iodide was used. Briefly, cells were washed twice in sterile water, suspended in PBS, and stained with 5 mg/mL propidium iodide (Sigma-Aldrich, Saint Louis, MO, USA) for 15 min in the dark at room temperature. Fluorescence pictures were taken using an Olympus BX-51 microscope equipped with a DP-72 digital camera and cell Sens Dimension software. Dead cells were red fluorescent (λex = 480 nm; λem = 520 nm). The data represent the mean values from three independent experiments. Statistical significance was assessed using Student’s *t*-test (*p* < 0.01).

### 2.16. Bacteria Strain, Growth Condition, and Antibacterial Activity

The antibacterial activity of the honey and extracts samples was assessed by monitoring the cell growth of a wide-use Escherichia coli DH5α strain (Thermo Fisher Scientific, Hennigsdorf, Germany). Bacterial cells were grown in a standard LB medium (LB Broth Lennox; BioShop, Burlington, Canada) without (control) or with tested honeys or extracts on a rotary shaker at 180 rpm. The growth was monitored using an Anthos 2010 type 17 550 microplate reader at 600 nm by measurements at 2 h intervals for 12 h. Each experiment was repeated at least three times. The experiments were carried out at optimal for bacteria 37 °C. The optical density of the culture was monitored every 2 h, at 600 nm wavelength.

### 2.17. Statistical Analysis

All analyses were performed in triplicates. The results are presented as mean ± standard deviation. Significant differences between data obtained for tested samples were assessed using Tukey’s test (*p* = 0.05) or Student’s *t*-test (*p* = 0.01). The correlations between the results were expressed by the Pearson correlation coefficient. Principal component analysis (PCA) and cluster analysis were carried out to group the test samples according to the parameters tested. All calculations were performed using Statistica 13.1 software (StatSoft, Inc., Tulsa, OK, USA).

## 3. Results and Discussion

### 3.1. Physicochemical Parameters of Honey Quality

Firstly, the physicochemical parameters were analyzed for 10 goldenrod honeys to assess their quality regarding obligatory UE standards (Table S1). It can be concluded that the analyzed samples meet the requirements of the EU Directive of 2014 [17], which clearly indicates the quality requirements for varietal honeys. It was found that goldenrod honeys are characterized by a varied water content, which was on average 17.97% but did not exceed 19.55%, which fulfills guidelines (below 20%). Such moisture content is specific for Polish nectar honeys and has been reported earlier [18,19].

The mean pH value of the tested honeys was 4.27 and was higher than the results for goldenrod honeys obtained by Ratiu et al. [20], where the values ranged from 3.31 to 3.67. The inverse relationship was found by analyzing the acidity. Studied goldenrod honeys had lower values (13.95–32.55 mEq/kg) compared to foreign goldenrod honeys, where the parameter was in the range of 42.7–49 mEq/kg [20]. This feature can be specific for Polish nectar honey. Tomczyk et al. [19], examining Polish varietal honeys, found the lowest mean acidity values for acacia (16.1 mEq/kg), and the highest for multiflorous honey (37.0 mEq/kg).

Electrical conductivity can help distinguish nectar from honeydew honey. The EU Directive [17] indicates that the conductivity of nectar honeys cannot be lower than 0.2 mS/cm and not higher than 0.8 mS/cm. In the analyzed goldenrod honey samples, the average value of the conductivity was in the range of 0.139–0.592 mS/cm, and for four samples, the value of the tested parameter was below the limit, and the obtained mean value of the conductivity of 0.263 mS/cm is lower compared to the results obtained by Ratiu et al. [20], where the tested parameter for goldenrod honeys ranged from 0.331 to 0.669 mS/cm.

Tested honey samples were characterized by proper sugar profile (data not shown) and 5-hydoxymethylfurfural (HMF) content not exceeding obligatory limits (Table S1).

Tested samples were strongly diversified in the terms of α-amylase activity, determined as diastase number (DN). The values of DN were found from 11 to 22 (Table 1). However, all of the tested honeys are within the applicable limits amounted 8 DN [17]. As the diastase activity is a known indicator of the biological activity of honey and its overheating, samples no. 1, 3, 5 and 10 can be assumed as the most active honeys. The soluble protein content ranged from 22 to 89 mg/100 g, which are typical values for Polish light honeys—19.09–133.18 mg/100 g, for acacia, linden and multifloral honey [13].

**Figure 1:**
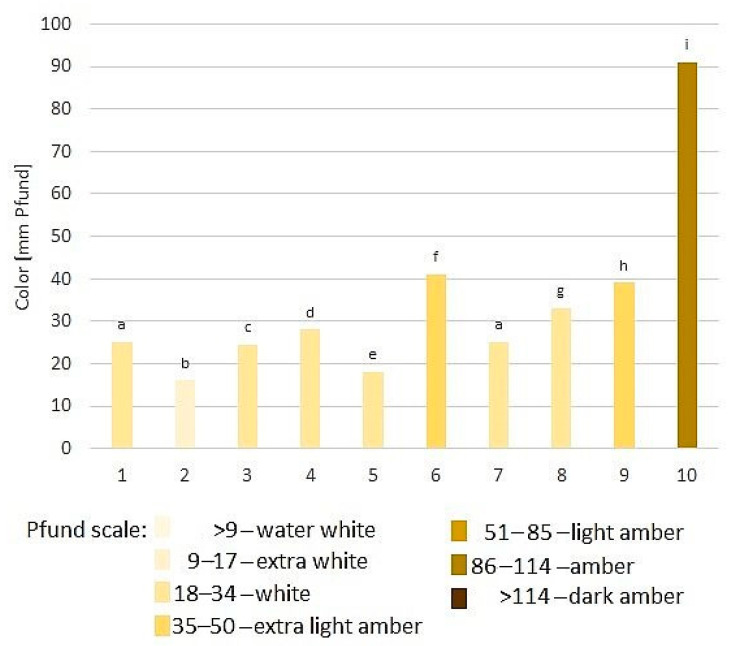
Color analysis of goldenrod honey samples (1–10). Different letters above the bars indicate significant differences (*p* = 0.05).

Honey color is a parameter that is visually evaluated by beekeepers in Poland, however, we measured it by colorimeter (Figure 1). The tested samples of goldenrod honeys belonged to light varieties except for sample 10, which showed a slightly darker shade, and it was visible also in higher values of color parameters determined for this sample. Goldenrod honeys from three countries, studied by Czigle et al. [2] were classified as water white to light amber for Slovak and Hungarian samples, whereas honeys from Poland had a color in the range of 0.30 to 7.37 mm Pfund, which puts them in the water white group [2].

### 3.2. Protein Composition

**Figure 2:**
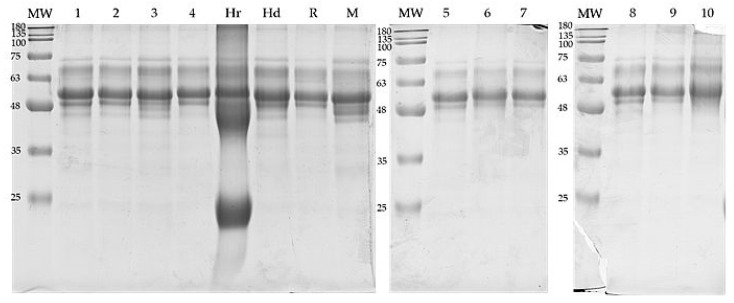
Protein profiles of analyzed goldenrod honey samples in comparison to other selected honeys obtained using SDS-PAGE. Abbreviations: 1–10—goldenrod honeys, Hr—heather honey, Hd—honeydew honey, R—rapeseed honey, M—multifloral honey, MW—BlueEasy Prestained Protein Marker.

The protein profiles of tested goldenrod honeys were obtained by SDS-PAGE analysis and compared to other varietal honeys (Figure 2). The presence of specific bands in the range between 50 and 75 kDa was found as specific for goldenrod honey, whereas all honeys contain protein fractions at around 60, 70 and 75 kDa.

**Figure 3:**
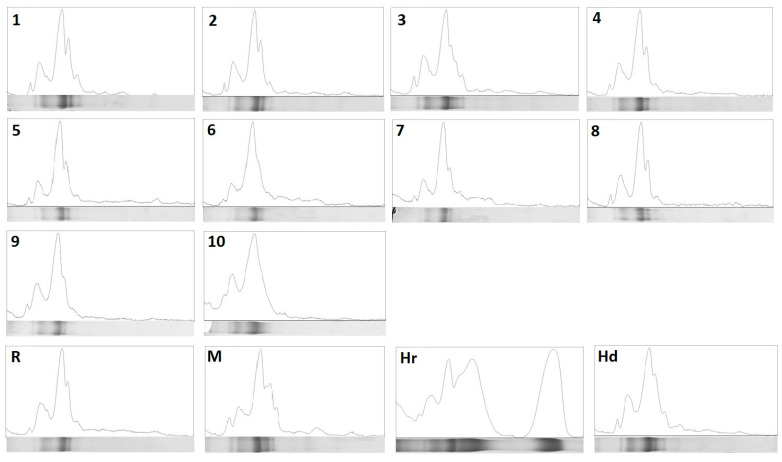
Detailed analysis of particular SDS-PAGE protein profiles obtained for analyzed goldenrod honeys (1–10) and four reference honeys (Hr—heather, Hd—honeydew, R—rapeseed, M—multifloral) in ImageJ software.

The heather honey used as reference differs markedly from the rest with distinct fractions at 40 kDa and 25 kDa. All samples, except heather honey and test samples 6 and 10, have a band at a height of about 48 kDa. However, sample no. 3 as well as honeydew and multifloral honey have a specific arrangement of bands in the range of 40–48 kDa visible on the gels. These differences are best seen in profiles generated in ImageJ (Figure 3). Comparing the course of obtained graphs, the specific fingerprint for goldenrod honey can be pointed as a typical pattern as in the case of sample no. 1, 2, 4 and 8. Based on distinct profiles observed for samples 3, 6 and 10, it can be concluded that the variety is not pure, and the tested honey may originate from various plant sources and even contain an admixture of honeydew. We obtained similar profiles earlier for varietal honeys, although no clear peaks in the range of 40–48 kDa were observed in the case of the multifloral honey analyzed at that time [16]. Previously, the differences in the band pattern on SDS-PAGE gels between the different types of honey were demonstrated by Baroni et al. [9]. They also linked characteristic proteins present in plant pollen to those shown on gels for honey samples. Very similar profiles for varietal honeys were obtained by Muresan et al. [21], with repetitive proteins in the mass range of 45–85 kDa.

### 3.3. HPTLC Analysis

**Figure 4:**
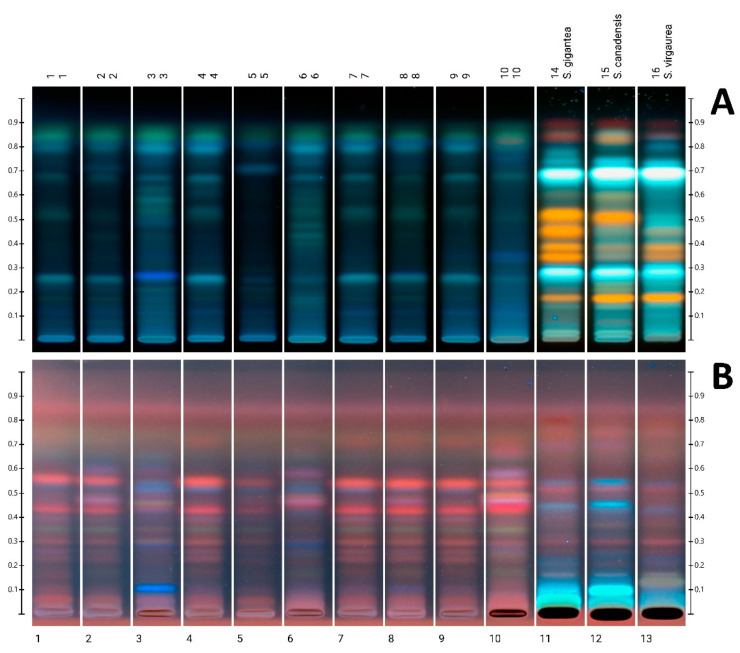
HPTLC plates images: (**A**)—developed with eluent system A, derivatized with NP/PEG reagent, (**B**)—developed with eluent system B, derivatized with *p*-anisaldehyde reagent, Track 1–10: goldenrod honey, track 11: *S. gigantea*, track 12: *S. canadensis*, track 13: *S. virgaurea*.

HPTLC phenolic profiles obtained with two mobile phase systems as well as two different derivatizing agents (Figure 4) are similar for most tested samples, allowing the specification of a typical profile for goldenrod honey and its authentication. The characteristic, repeating pattern of bands is visible, especially in the case of derivatization with *p*-anisaldehyde. In the case of samples 3, 6 and 10, the profiles are significantly different. In the case of sample 10, it is possible to infer an admixture of honeydew, which is manifested by characteristic additional pink bands at Rf approx. 0.48 and 0.6. This pattern is similar to the one we observed earlier for honeydew honey samples [16]. These pink bands are also present, although less intense, in sample 6. The obtained profiles seem to correlate with the higher content of phenolic compounds in the three above-mentioned samples and their stronger antioxidant properties which was particularly observed in sample 10 where the admixture of honeydew could explain the strongest antioxidant properties (see Section 3.4). In honey number 3, additional intense bands are visible. Figure 4A shows blue ones at Rf = 0.26 and a series of blue-green between 0.45 and 0.7. A similar series of bands are clearly visible in sample 6. Figure 4B shows an additional blue band at Rf = 0.11 in sample 3, observed by us earlier in the rape honey sample [16].

Since three of the samples (3, 6 and 10) differ from the others in terms of polyphenol profiles, we decided to evaluate the flower extract profiles of three species of goldenrod to check whether the differentiation of the honey profiles is not related to another species of *Solidago* as a honeybee flow. The extracts of the individual species differ in particular in the Rf region between 0.30 and 0.55 (Figure 4A), where the bands derived from flavonoid glycosides, mainly derivatives of quercetin and/or luteolin, were identified. *Solidago gigantea* flowers contain four distinct glycosides, whereas in *S. canadensis*, only one with the highest Rf was present in large amounts, and in *S. virgaurea*, two with lower Rf (0.34 and 0.38), were identified as quercitrin and hyperoside. Rutin was present in all three species (Rf = 0.18). In addition, in *S. canadensis*, extracts from the bands of flavonoid aglycones, quercetin and kaempferol (Rf = 0.82 and 0.84, respectively) were detected. Derivatization with *p*-anisaldehyde revealed an additional gray-green band in the *S. virgaurea* extract at Rf = 0.13, possibly from some phenolic acid. Based on the comparison of the tracks from honey and goldenrod flower extracts, it can be theorized that the two most intense bands present in all three species of flowers (Rf = 0.28 and 0.69, Figure 4A) are most likely derived from certain phenolic acids, and are also present in most of the analyzed honey samples.

Among the polyphenols mentioned as present in goldenrod honey are mainly phenolic acids: gallic, 4-hydroxybenzoic, *p*-coumaric and ferulic [1]. Flavonoids have also been identified, mainly chrysin, galanagin, pinocembrin, kaempferol, quercetin and luteolin [22]. In flowers of various species of goldenrod, flavonoids were identified: mainly quercetin glycosides, and also aglycones: quercetin, kaempferol andisoramnetin [23,24,25]. In addition, in *S. virgaurea* flowers, myricetin, naringenin, genistein and pinocembrin were identified [1]. Among phenolic acids, chlorogenic, caffeic and ferulic acids were mainly found in the flowers of *S. gigantea* and *S. canadensis* [23,25] and gallic, 3,4-dihydroxybenzoic and vanillic acids were found in the flowers of *S. virgaurea* [1]. The quoted literature data indicate a certain variability in the polyphenol profile between individual goldenrod species, with *S. gigantea* and *S. canadensis* being more similar to each other than to *S. virgaurea*. Similar polyphenol profiles were obtained by HPLC for *S. canadensis* and *S. gigantea* by Zekič et al. [26].

To confirm the richest polyphenol profile obtained for *S. gigantea*, the total content of phenols, flavonoids and antioxidant activity of the extracts of three species of goldenrod (flowers and leaves) were analyzed (Table S2). The obtained results also indicate that this species is the richest in phenolic compounds, which is reflected in its antioxidant capacity. Moreover, the data shows a high correlation (TPC vs. FRAP: r = 0.991, TPC vs. DPPH: r = 0.972, TPC vs. CUPRAC: r = 0.948). Literature data also indicate this goldenrod species as richer in polyphenols and antioxidants [27,28,29]. Based on provided literature data and own results, some species–specific differences were found; however, no obvious polyphenol markers were identified that could indicate which species of goldenrod is the source nectar for the analyzed honeys.

### 3.4. Antioxidant Properties of Goldenrod Honey

The goldenrod honeys were characterized in terms of the total content of phenols and flavonoids. The results obtained for ten honey samples are summarized in Table 2.

The content of phenolic compounds ranged from 15.77 to 50.69 mg GAE/100 g, whereas sample 10 significantly differed from the others, for which TPC did not exceed 31.75 mg GAE/100 g. Flavonoids constituted a small percentage of phenolic compounds—on average between 0.2 and 0.4 mg QE/100 g, except for sample 10, containing 0.77 mg QE/100 g. According to literature data, goldenrod honey belongs to honeys with moderate phenolic content. For Polish goldenrod honey, this range was 11.29–21.03 [1], 28.4 to 96.6 [30] and even 250 mg GAE/100 g [31]. The content of flavonoids determined by Jasicka-Misiak et al. [1] was found at a similar level: 0.93–1.41 mg QE/100 g honey. It is well known that light honeys are characterized by a lower phenolic content and lower antioxidant activity than dark honeys [32,33]. The data for the antioxidant capacity expressed by the three methods show little differentiation of the tested samples. The content of phenols and flavonoids is strongly correlated (r = 0.986). The FRAP reducing power also strongly correlates with the content of polyphenols (r = 0.989 and 0.969 for TPC and TFC, respectively). There are three of them: 3, 6 and 10, and regardless of the method used, they achieved the highest results. Similar data for Polish honeys were obtained earlier. The FRAP reducing ability of Polish goldenrod honeys ranged from 60.57 to 235 µmol TE/100 g [30] and the antiradical activity (DPPH) was 44.9 µmol TE/100 g [31]. In the latest study comparing goldenrod honeys from Poland, Slovakia and Hungary, Polish honeys were the weakest among the compared [2]. This proves the high variability even within one honey variety, due to climatic conditions and the exact composition of the flora.

### 3.5. Statistical Analysis

**Figure 5:**
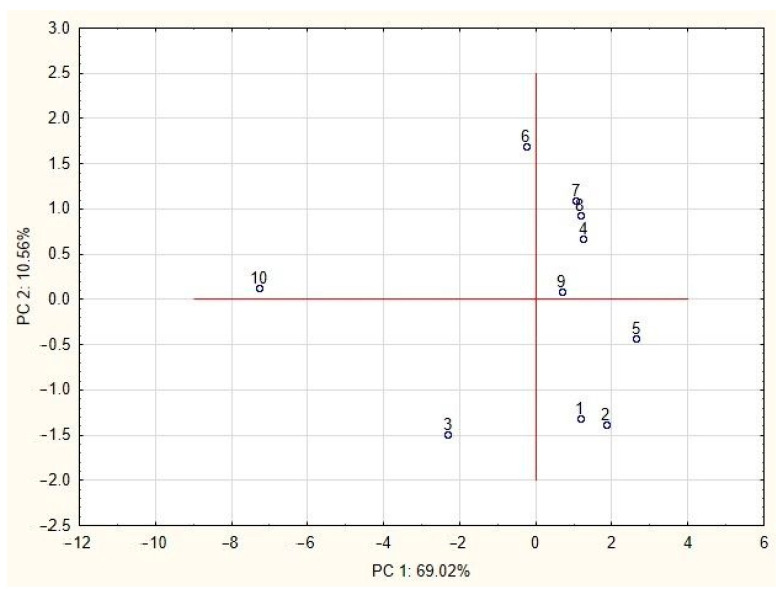
PCA score plot of tested goldenrod honey samples (1–10).

To confirm the hypothesis resulting from the analysis of the presented results: samples 3, 6 and 10 are not typical goldenrod honeys, and a multivariate statistical analysis was performed. In the first step, principal components analysis (PCA) was used, by which the samples were grouped using the projection of cases onto the factor plane (PC 1 × PC 2) (Figure 5). Samples 3 and 10 clearly differ from the others; also sample 6 can be considered as different, located on the negative side of the PC 1 principal component. Among the honey samples considered typical for goldenrod, two groups can be distinguished: samples 4, 6, 7 and 9 as well as 1, 2 and 5.

**Figure 6:**
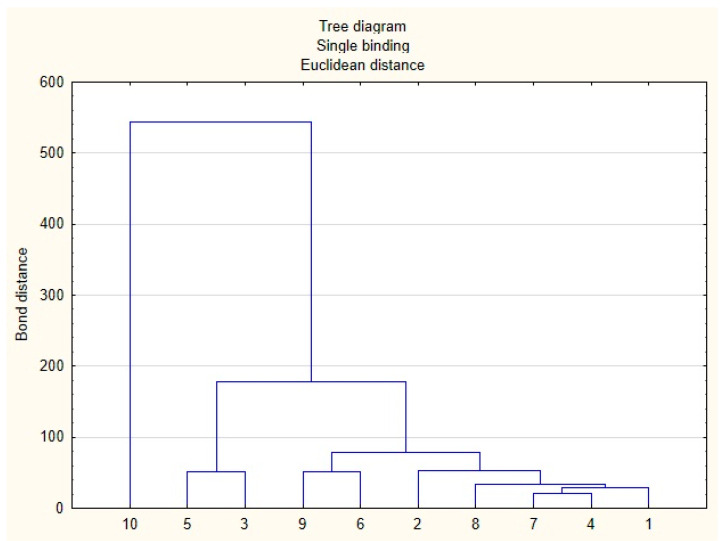
Tree diagram based on the average values of tested parameters for goldenrod honey samples (1–10).

A cluster analysis was also performed, taking into account the tested honey parameters (Figure 6). The analysis showed the greatest differences for sample 10, which is confirmed by the highest bond distance. For samples 3 and 6, some similarities were demonstrated with other samples considered typical, 5 and 9, respectively, which may indicate the presence of admixtures to the base honey, which significantly changes its properties. The highest degree of similarity was shown by samples 1, 2, 4, 7 and 8, which was presented earlier with the use of SDS-PAGE protein and polyphenolic HPTLC profiles.

### 3.6. Antimicrobial Activity

**Figure 7:**
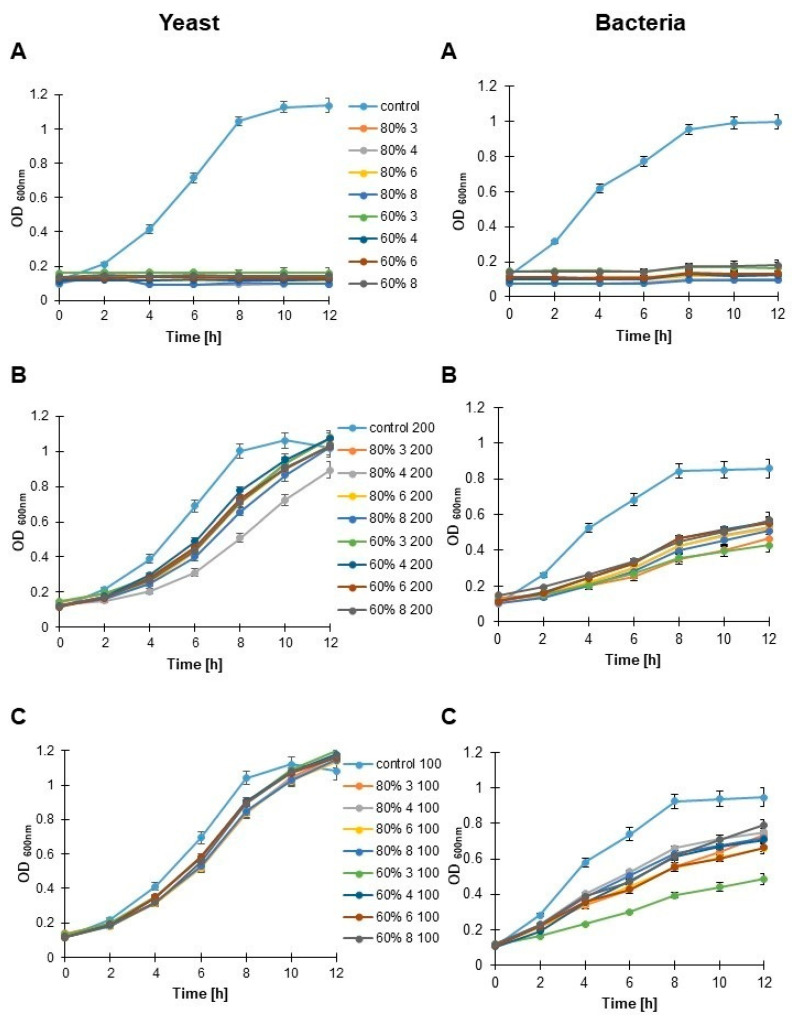
The effect of increasing doses of selected goldenrod honey samples (3, 4, 6 and 8) on budding yeast (**left**) and bacterial (**right**) growth. (**A**)—undiluted honey samples, (**B**)—samples in dilution 1:5 (200 mg/mL), (**C**)—samples in dilution 1:10 (100 mg/mL).

Various types of honey have been tested for their antibiotic or bacteriostatic activity for a long time, increasing our knowledge of the subject. Therefore, the next purpose of this study was to enhance general knowledge about goldenrod honey’s bacteriostatic (against *Escherichia coli*) and fungicidal (against budding yeast) activities using four representative samples: 4 and 8 (with typical protein and HPTLC profiles) and 3 and 4 (with distinct features). First, we compared the growth rates of both yeast and bacteria cells. As can be seen in Figure 7A, 80% and 60% honeys completely stopped the growth of microbial cells, respectively. Then we diluted the solutions of the analyzed honeys in microbiological media in the ratio of 1: 5. As shown in Figure 7B, bacterial growth was much more restricted than yeast’s. The most powerful inhibitory effect on yeast was found with 80% honey solutions 4 and 8. During the first 6 h, we found no significant differences in the effect of honey on bacteria. A slightly higher bacteriostatic effect was observed in the second part of the experiment with 80% and 60% of honey 3 (Figure 7B). Then, we diluted the honeys 1:10. Compared to the untreated honey control, yeast cell growth was only slightly inhibited (Figure 7C). In turn, this dilution significantly inhibited bacterial growth, especially in honey solution 3 (Figure 7C). In summary, we observe concentration-dependent inhibition of microorganism growth, especially bacteria.

**Figure 8:**
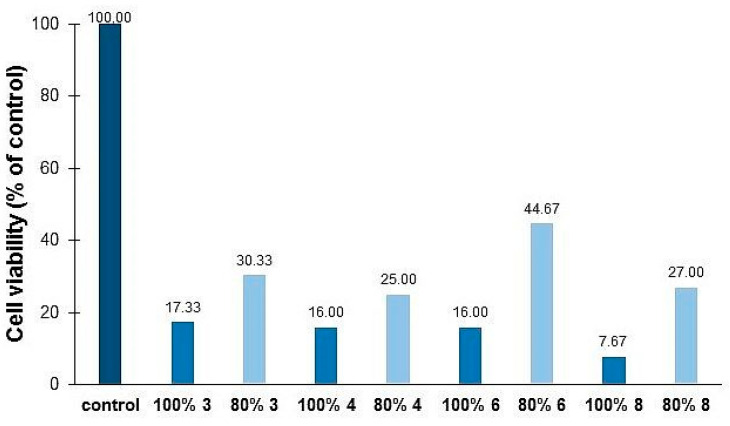
The fungicidal activity of selected goldenrod honey samples (no. 3, 4, 6 and 8).

We then tested whether goldenrod honey and its 80% solution affected yeast viability. The dead cells were determined by fluorescently detecting the number of propidium iodide-labeled dead cells after incubation with honey and honey solution for 4 h at 28 °C. Figure 8 shows that the analyzed honeys do not cause 100% death of yeast cells, indicating that the arrest of growth is caused by the arrest of the cell cycle rather than the death of cells. Honey 8 displayed the highest fungicidal activity (7.67%). Meanwhile, no difference in survival was observed between honeys 3, 4 and 6. It is interesting to note that yeast cells treated with 80% honey solution survived at a rate of 44.67%. In conclusion, goldenrod honey does not cause cell death in all yeast species. It is necessary to conduct further research to identify a yeast cell population that is resistant to honey. The age of the cell and sensitivity of young cells possibly play a role here.

It is well known that the components of honey show different activities against various microorganisms. Honey activity is dependent on the plant species, the weather conditions and the natural properties of the nectar [34]. The use of honey as a drug for the treatment of disease dates back to 2000 BC. Aristotle first described honey as “good for sore eyes and wounds” [35]. However, only a few decades ago, the first scientific reports confirmed the antibacterial and antifungal properties of honey. It has been shown many times that honey has strong antimicrobial properties against most fungi and bacteria that cause wound and surgical infections. The microorganisms were grown in both aerobic and anaerobic conditions. As was shown, growth inhibition was complete in the media containing 100%, partial in media containing 50% and no inhibition was produced by 20% of honey. Pure honey was therefore an ideal wound dressing agent, at least on a topical basis, for surgical infections, burns and wound infections [36].

Currently available agents are no longer effective against many dermatophytes. It seems that medicine, especially dermatology, has an urgent need for new effective antifungal agents suitable for the treatment of superficial skin infections. A recent study showed that Agastache honey had fungicidal activity against dermatophytes and yeast *Candida albicans* at 40% concentration [37]. Some previously report show that honey samples from different floral sources significantly inhibit the growth of few yeast species e.g., *Candida albicans, C. krusei, C. glabrata* and *Trichosoporon* spp. Inhibition of growth tests was found to depend on the type and concentration of honey and the pathogen tested [38]. In our previous study, we reported that honeys enriched with chokeberry fruits had anti-bacterial and anti-viral properties [39]. Here, we showed that goldenrod honey has antifungal properties that significantly inhibit yeast growth, but do not result in the death of the entire population. There are likely to be two factors contributing to this: the hydration of the honey and the goldenrod flowering in autumn. It is possible that some species of bacteria and yeast may ferment this honey because it has no antiseptic properties. Apparently, these data are crucial for beekeepers, for example, in the context of honey dehydration or honey fermentation in hives.

## 4. Conclusions

Protein and polyphenols profiling was used to verify the authenticity of 10 honey samples declared by beekeepers as goldenrod. The conclusions from the obtained varietal fingerprints (specific for 70% of the evaluated samples) were supported by standard physicochemical parameters and antioxidant activity of honey as well as PCA and cluster analysis. For selected samples, the antimicrobial effects against model bacteria and yeast strains were verified. HPTLC polyphenolic profiles comparison for honey and extracts of three *Solidago* species did not allow to determine of an exact botanical source of honey. It was found that SDS-PAGE and HPTLC assays allow for obtaining the protein and polyphenol fingerprints for goldenrod honey authentication. Final recognition of these methods requires confirmation for other honey varieties.

## Figures and Tables

**Table 1:** Protein content and diastase number (DN) of tested honey samples.

Sample	1	2	3	4	5	6	7	8	9	10
Soluble protein content [mg/100 g]	38.11 ± 7.97 ^a^	22.68 ± 1.28 ^a^	38.91 ± 8.35 ^ab^	39.01 ± 8.98 ^ab^	35.39 ± 9.08 ^ac^	60.79 ± 11.55 ^bd^	51.72 ± 14.25 ^bc^	75.31 ± 8.98 ^d^	57.16 ± 6.42 ^bcd^	88.92 ± 10.27 ^d^
Diastase number (DN)	25.96 ± 0.42 ^a^	16.92 ± 1.15 ^b^	24.40 ± 0.68 ^a^	12.20 ± 0.85 ^cd^	17.68 ± 1.25 ^b^	10.68 ± 0.85 ^c^	11.41 ± 0.00 ^cd^	15.09 ± 0.42 ^be^	13.62 ± 0.42 ^de^	22.10 ± 0.85 ^f^

Means sharing the same letter in a row are significantly different at *p* = 0.05.

**Table 2:** Total phenolics and flavonoids content as well as antioxidant properties of goldenrod honeys.

Sample	TPC [mg GAE/100 g]	TFC [mg QE/100 g]	DPPH [μmol TE/100 g]	FRAP [μmol TE/100 g]	CUPRAC [μmol TE/100 g]
1	17.36 ± 1.69 ^a^	0.31 ± 0.02 ^a^	22.81 ± 12.62 ^a^	19.74 ± 1.74 ^a^	302.47 ± 23.49 ^a^
2	18.06 ± 0.75 ^a^	0.26 ± 0.01 ^a^	24.66 ± 8.25 ^a^	21.93 ± 2.31 ^a^	379.37 ± 54.01 ^ac^
3	31.75 ± 0.91 ^b^	0.48 ± 0.02 ^b^	29.27 ± 16.94 ^a^	62.28 ± 2.74 ^b^	625.45 ± 46.99 ^b^
4	22.22 ± 1.89 ^c^	0.33 ± 0.01 ^ad^	25.58 ± 15.07 ^a^	33.11 ± 0.38 ^c^	333.23 ± 35.52 ^ac^
5	15.77 ± 1.95 ^a^	0.20 ± 0.03 ^c^	15.44 ± 12.45 ^a^	19.74 ± 3.29 ^a^	640.83 ± 38.71 ^b^
6	25.50 ± 1.20 ^cd^	0.36 ± 0.01 ^d^	31.34 ± 14.21 ^a^	46.93 ± 1.00 ^d^	435.76 ± 44.40 ^c^
7	21.83 ± 0.69 ^c^	0.34 ± 0.02 ^ad^	21.43 ± 2.39 ^a^	33.55 ± 0.66 ^c^	317.85 ± 8.88 ^ac^
8	18.65 ± 1.64 ^ac^	0.30 ± 0.01 ^a^	20.28 ± 4.17 ^a^	23.46 ± 1.00 ^a^	297.34 ± 49.44 ^a^
9	27.78 ± 0.45 ^d^	0.38 ± 0.02 ^d^	20.74 ± 2.07 ^a^	43.20 ± 1.00 ^d^	451.14 ± 72.68 ^c^
10	50.69 ± 0.69 ^e^	0.77 ± 0.01 ^e^	39.18 ± 2.88 ^a^	96.93 ± 3.38 ^e^	1168. 87 ± 30.76 ^d^

GAE—gallic acid equivalents, QE—quercetin equivalents, TE—Trolox equivalents. Means sharing the same letter in a column are significantly different at *p* = 0.05.

## Data Availability

The data presented in this study are available in the article and Supplementary Material.

## Supplementary Materials

The following supporting information can be downloaded at: https://www.mdpi.com/article/10.3390/foods11162390/s1, Table S1: Physicochemical parameters of goldenrod honeys; Table S2: Polyphenols content and antioxidant capacity of *Solidago* spp. extracts.
