# Supplementary material for: SDS-PAGE Protein and HPTLC Polyphenols Profiling as a Promising Tool for Authentication of Goldenrod Honey
Source: Foods. 2022 Aug 9;11(16):2390. doi: 10.3390/foods11162390 (PMC9407375; doi:10.3390/foods11162390)
Supplement: Supplementary file 1 [file foods-11-02390-s001.zip › foods-1836125-supplementary.pdf]

Table S1. Physicochemical parameters of goldenrod honeys

	Water content [%]	pH	Acidity [mEq/kg]	Conductivity [mS/cm]	HMF content [mg/kg]
**1**	17.05 ± 0.65[a]	4.07 ± 0.005[a]	21.95 ± 0.00[a]	0.199 ± 0.0005[a]	20.09 ± 0.34[a]
**2**	19.55 ± 0.25[b]	4.10 ± 0.00[ad]	28.15 ± 0.05[b]	0.172 ± 0.000[b]	12.86 ± 0.24[b]
**3**	17.40 ± 0.10[ac]	4.68 ± 0.01[b]	24.70 ± 0.40[c]	0.418 ± 0.0005[c]	14.40 ± 0.83[b]
**4**	19.10 ± 0.20[b]	4.30 ± 0.00[c]	15.15 ± 0.15[d]	0.227 ± 0.001[d]	23.60 ± 0.26[c]
**5**	19.40 ± 0.40[b]	4.15 ± 0.005[ae]	13.95 ± 0.05[e]	0.139 ± 0.000[e]	20.16 ± 0.36[a]
**6**	17.00 ± 0.40[a]	4.28 ± 0.005[c]	17.95 ± 0.25[f]	0.178 ± 0.0005[f]	26.86 ± 0.56[d]
**7**	17.60 ± 0.30[ac]	4.23 ± 0.005[ce]	16.40 ± 0.40[g]	0.250 ± 0.0015[g]	23.02 ± 0.22[c]
**8**	18.05 ± 0.05[c]	4.20 ± 0.005[cde]	18.20 ± 0.80[f]	0.219 ± 0.0005[h]	4.80 ± 0.32[e]
**9**	18.05 ± 0.15[c]	4.22 ± 0.005[ce]	18.00 ± 0.10[f]	0.240 ± 0.0005[i]	20.72 ± 0.20[a]
**10**	16.55 ± 0.15[a]	4.60 ± 0.005[b]	32.55 ± 0.35[h]	0.592 ± 0.000[j]	30.51 ± 0.27[f]
**Applicable limits [EU Directive]**	max. 20%	-	max. 50	max. 0.8	max. 40

Means sharing the same letters in the column are significantly different at p=0.05

Table S2. Polyphenols content and antioxidant capacity of *Solidago* spp. extracts

Sample		TPC [mg GAE/100 g]	TFC [mg QE/100 g]	DPPH [µmol TE/100 g]	FRAP [µmol TE/100 g]	CUPRAC [mmol TE/100 g]
S. virgaurea	L	77.03 ± 5.51[a]	25.80 ± 0.66[a]	206.49 ± 9.80[a]	412.50 ± 22.79[a]	4.44 ± 0.98[a]
	F	44.94 ± 2.63[A]	18.40 ± 0.53[A]	112.12 ± 3.21[A]	208.88 ± 17.56[A]	1.97 ± 0.14[A]
S. canadensis	L	49.26 ± 2.63[b]	21.09 ± 0.62[a]	95.98 ± 4.60[b]	215.90 ± 6.09[b]	2.06 ± 0.03[b]
	F	47.92 ± 0.79[A]	27.53 ± 0.84[B]	120.87 ± 10.60[AB]	222.26 ± 5.12[A]	2.09 ± 0.15[A]
S. gigantea	L	86.76 ± 4.66[a]	55.95 ± 3.35[b]	226.19 ± 4.18[c]	435.75 ± 20.79[a]	3.95 ± 0.29[a]
	F	49.26 ± 1.83[A]	37.86 ± 0.68[C]	131.01 ± 2.42[B]	213.16 ± 11.31[A]	1.92 ± 0.04[A]

L – leaf, F – flower. Means sharing the same letter in the column (lowercase for leaves and uppercase for flowers) are significantly different at p=0.05
